# Knowledge and Willingness toward Vaccination among Pregnant Women: Comparison between Pertussis and Influenza

**DOI:** 10.3390/ijerph192114082

**Published:** 2022-10-28

**Authors:** Feng Jiang, Ning Tang, Yuanxue Gao, Jun Feng, Ying Wang, Bin Qu

**Affiliations:** 1 Institute of Expanded Programme on Immunization, Guizhou Provincial Center for Disease Control and Prevention, Guiyang 550004, China; 2 School of Public Health, Fudan University, Shanghai 200032, China; 3 Zunyi Prefectural Center for Disease Control and Prevention, Zunyi 563000, China

**Keywords:** pertussis, influenza, vaccination, pregnancy

## Abstract

Background: Our study sought to characterize the knowledge and willingness levels regarding vaccinations against pertussis and seasonal influenza (influenza) among pregnant women in Guizhou province, China, which have previously been unclear. Methods: In total, 11 hospitals that carried out obstetrics and antenatal examination services were randomly included in the target organizations, and 564 questionnaires completed by the pregnant women were collected and analyzed in Guizhou province. The questionnaires contained questions addressing awareness and knowledge of pertussis and influenza, willingness to be vaccinated at different life stages, and the basic statuses of subjects. A two-paired McNemar test was used to compare the knowledge levels on pertussis and influenza. A Friedman test was used to compare the willingness to be vaccinated at different life stages. To explore the factors influencing knowledge levels, a chi-square test and binary logistic regression were used with stepwise backward regression. Results: In total, 11.9 percent of the pregnant women had received influenza vaccines in the year prior to their pregnancy in Guizhou province. The pregnant women had poorer knowledge of pertussis than of influenza. Given a vaccine was available, the willingness of pregnant women to partake in the following vaccination-related actions could be ranked, from highest to lowest: free vaccination of babies, recommend vaccination to family members, postpartum vaccination, vaccination of babies at mothers’ expense, and vaccination during pregnancy. Knowledge levels played different roles in the women’s willingness to receive vaccinations at different life stages. Common knowledge of pertussis and influenza played a limited role in the willingness to receive maternal vaccinations. Among the pregnant women, the factors influencing the low levels of pertussis knowledge were occupation as nonmedical-institution staff, lower educational level, pregnancy stage past the first trimester, and not bearing children; for influenza, the factors were occupation as nonmedical-institution staff, lower educational level, denial of pregnancy-induced disease, and lower monthly household income per capita. Conclusions: Pregnant women have poorer levels of knowledge on pertussis than influenza, whereas there was no significant difference in their willingness to be vaccinated against these conditions. Health education on pertussis should be strengthened and we called for vaccines given at birth.

## 1. Introduction

Pregnant women and young children, especially young infants, are at greater risk of severe disease or complications when infected with seasonal influenza (influenza) and pertussis (whooping cough) [1]. Influenza and pertussis spread easily from person to person, mainly through droplets produced by coughing or sneezing [2]. Vaccination of pregnant women is likely to be the most cost-effective additional strategy for preventing pertussis in infants too young to be vaccinated, and it appears to be more effective and favorable than cocooning [3,4]. The World Health Organization (WHO) has recommended vaccination against influenza in pregnant women since 2005 [5]. In 2014, the Chinese Advisory Committee on Immunization Practice updated the guidelines for the application of the seasonal influenza vaccine (SIV), which recommended SIV vaccination of pregnant women as a high-priority group [6]. The DTP (combined diphtheria, tetanus, and pertussis) vaccine has been included in the China National Immunization Program since 1978, and now, the immunization schedule of the DTaP (combined diphtheria, tetanus, and acellular pertussis) vaccine is at 3, 4, 5, and 18 months for children [7,8]. However, in China the pertussis-containing vaccines were not applicable to teenagers, adults, and pregnant women [9]. In 2021, expert consensus based on the China Pertussis Initiative suggested to improve the immunization program and strategy of the pertussis vaccine in China, including by exploring how to carry out booster immunization for school-age children, adolescents, adults, and pregnant women on the basis of existing immunization schedules [10]. Vaccination against influenza and pertussis is routinely recommended during pregnancy in several countries [11,12]. This prevention strategy of maternal vaccination has hardly been carried out in China for either influenza or pertussis, and guidelines specifically for pregnant woman are usually not included in the information accompanying the vaccines [13,14].

In 2017, the greatest number of cases of pertussis were reported in India (23,766), Germany (16,183), Australia (12,114), and China (10,390) [15]. In China, the pertussis incidence rate appears to be going up, especially in the population of children less than one year of age, and about 70% of children do not start the DTaP vaccination schedule or have not completed primary immunization [9]. Guizhou province is an economically underdeveloped province of China, located in the southwestern area. In recent years, there have been frequent outbreaks of influenza and family-scattering cases reported in this area [16,17,18]. According to influenza surveillance sentinel data, the positive detection rate for influenza virus nucleic acid was as high as 30.79% in the reported cases of influenza-like illness in the influenza surveillance years of 2017–2018 [19]. Meanwhile, the pertussis incidence rate showed a significant upward trend, and the incidence rate in 0-year-old children was highest, reaching 91.2/100,000 between 2017 and 2019 in Guizhou province. Among the reported pertussis cases, 80% of whooping cough patients had not completed the primary series of three doses of DTaP vaccination, mainly because they were too young at the time of the onset month, namely, below the age of 5 months that is set for receiving the primary three doses in the national recommended immunization schedule of China [20]. The vaccines against influenza and pertussis given at birth are not applicable until this age. To protect infants, especially in the first few months of life, from influenza and pertussis, maternal vaccination is optimal.

Currently, the influenza vaccine is not included in the National Immunization Program, and it is optional and self-funded for the populations in Guizhou province, China. In recent years, the overall coverage rate of the SIV has risen in some areas in China [21,22], but it is still low in Guizhou province. In the 2020–2021 influenza season, the SIV vaccination rate of children aged 6 months to 5 years in Guizhou province was 16.27%, and the influenza vaccination rate of elderly people aged 65 years and over was 2.15% [23,24]. Recently, a survey showed that people aged 18-65 in mainland China had low knowledge levels on influenza and influenza vaccination [25]. Additionally, to our knowledge, pertussis knowledge was also limited even among healthcare workers [26]. However, the knowledge status is unknown amongst pregnant women, and there is no comparison of knowledge levels between influenza and pertussis and the role this plays in the willingness to vaccinate.

To explore vaccination strategies against pertussis and influenza, the vaccination attitudes and choices of pregnant women require special attention. Thus, a survey was conducted among pregnant women in Guizhou province from January to February 2022 to improve understanding of and address gaps in their knowledge. The aims of this study were: (1) to investigate disease common knowledge and attitudes toward pertussis and influenza vaccinations among pregnant women; (2) to explore the role of disease common knowledge in the willingness to receive vaccinations; and (3) to identify the factors influencing these knowledge levels.

## 2. Methods

### 2.1. Study Institutions

The information from the medical institutions in Guizhou province was obtained from the standard coding management system of China’s disease prevention and control information system. In total, 484 general hospitals, maternal and child healthcare hospitals, and traditional Chinese medicine (general) hospitals are registered under the health administration departments in Guizhou province. Of these, 15 hospitals were randomly selected through the method of simple random sampling using SPSS 22.0 software (IBM Corp. in Armonk, NY, USA). After a preliminary investigation of the medical institutions’ service content, 11 hospitals were included in the target organizations that carried out obstetrics and antenatal examination services. Among the 11 hospitals, 5 were state-owned and 6 were private, 1 was a tertiary medical institution, 5 were secondary hospitals, and 5 were first-level and below medical institutions. The eleven hospitals are located in the nine counties of Guizhou province, including five counties in the north (counties: Daozhen, Renhuai, Chishui, Hezhang, Qixingguan) and four counties in the center and south (counties: Xiuwen, Guanling, Kaili, Xingyi).

### 2.2. Study Subjects

From 20 January to 9 February 2022, the survey was carried out in the 11 hospitals, and pregnant women who visited the gynecology and obstetrics clinics were included in the survey with their informed consent. Meanwhile, the survey questionnaire was administered to the electronic contact groups of pregnant women in the hospitals. The inclusion criteria were women undergoing pregnancy, participation in this study for the first time, and informed consent. The exclusion criteria were an incorrect answer to a choice question (for example, for “What holiday is 1st October in China?”, the right answer is National Day) and other obvious errors in demographic information. Approval for the protocol was obtained from the Ethics Committee of the Guizhou Provincial Center for Disease Control and Prevention.

### 2.3. Survey Questionnaire and Questions

The survey questionnaire was self-developed. First, it was modified through pre-experimental tests on six women who were pregnant or new mothers. Second, it was tested on participants in a secondary hospital and a private hospital, where 59 eligible questionnaires were received. According to the above pilot tests, minor changes were made and those results were not included in the sample of the survey. 

The questionnaire collected the following data items: (1) Common knowledge about pertussis and influenza, including six items: ever heard of it; it is infectious; main source of infection; main mode of infection; main clinical manifestations; preventable vaccine. (2) Willingness to be vaccinated against pertussis and influenza at different life stages, including: vaccinate during pregnancy; vaccinate baby at own expense; vaccinate postnatally; recommend family households to get vaccinated; give baby free vaccination. (3) The basic statuses of subjects, including: birthday; gestational weeks; bearing children; educational level; occupation; location city/county name; registered residence type; ethnicity; pre-existing diseases; pregnancy-induced diseases; monthly household income per capita; vaccination history in the year before pregnancy; and so on.

### 2.4. Data Collection Tools

The survey questionnaire was established on the platform of Wen Juan Xing (Wen Juan Xing; Changsha Ranxing Information Technology Co., Ltd., Changsha, China). The pregnant women filled in the questionnaire by scanning the code using WeChat software (Tencent; Shenzhen, China) on their mobile phones. The data were collected by the Wen Juan Xing platform.

### 2.5. Knowledge Level

For comparison of the knowledge status between influenza and pertussis, the items of the questionnaire should stay comparable. Both influenza and pertussis are vaccine-preventable infectious diseases. We defined the common conceptions of infectious diseases as common disease knowledge to evaluate the knowledge levels for influenza and pertussis. The knowledge level was determined using six general questions on the disease and its vaccine (see Section 2.3. Survey Questionnaire and Questions). Each correctly marked item of the questionnaire added +1 to the sum score, whereas any incorrect one or an answer of do not know added 0. The knowledge level was calculated by the sum of the items’ scores. When comparing the influencing factors, we split the knowledge sum scores at the median numbers and divided them into low- and high-score levels. 

### 2.6. Sample Size

This research was a cross-sectional survey. The primary objective of this research was to obtain the knowledge level about influenza and pertussis among pregnant women. This cross-sectional survey sample-size calculation formula was used:

$$n=\frac{z_{\alpha }{}^{2}*\sigma^{2}}{\delta^{2}}$$

where *n* is the size of the sample, *α* is the probability of type Ⅰ error, *σ* is the standard deviation, and *δ* is the margin of error. According to the results of the pre-experimental tests, the mean (SD) of knowledge scores for pertussis was 3.44 (1.86), and the standard error was 0.24. We used *α* = 0.05, *σ* = 2.0, and *δ =* 0.2 in the formula, and the calculated sample size was 385 cases. Considering the invalid questionnaires and the losses during the survey, the sample size was expanded by 20%, and thus, to *n* = 462. The upward rounding sample size was 500.

### 2.7. Data Statistics

Categorical variables were described as proportions, and continuous variables were described as mean (SD) or median (IQR). To compare the distribution of two categorical data and three categorical data among two-paired samples, the McNemar test and Wilcoxon signed-rank test were used, respectively. Additionally, the Friedman test was used to compare categorical distribution among multiple paired samples. To explore the correlation between knowledge levels and vaccination willingness, Spearman’s correlation analysis was used. To explore the factors influencing the knowledge levels, the chi-square test and binary logistic regression were used with stepwise backward regression (*α* In = 0.05, *α* Out = 0.10). Depending on the univariate analysis results, variables with *p* ≤ 0.1 were chosen mainly for the multivariate analysis. To understand how the factors changed when considering only pregnant women working in nonmedical institutions, a subgroup analysis among the pregnant women working in nonmedical institutions was carried out using binary logistic regression as above. Data were analyzed by SPSS 22.0 software. Any *p*-values < 0.05 (bilateral) were considered to indicate statistically significant differences.

## 3. Results

**Figure 1:**
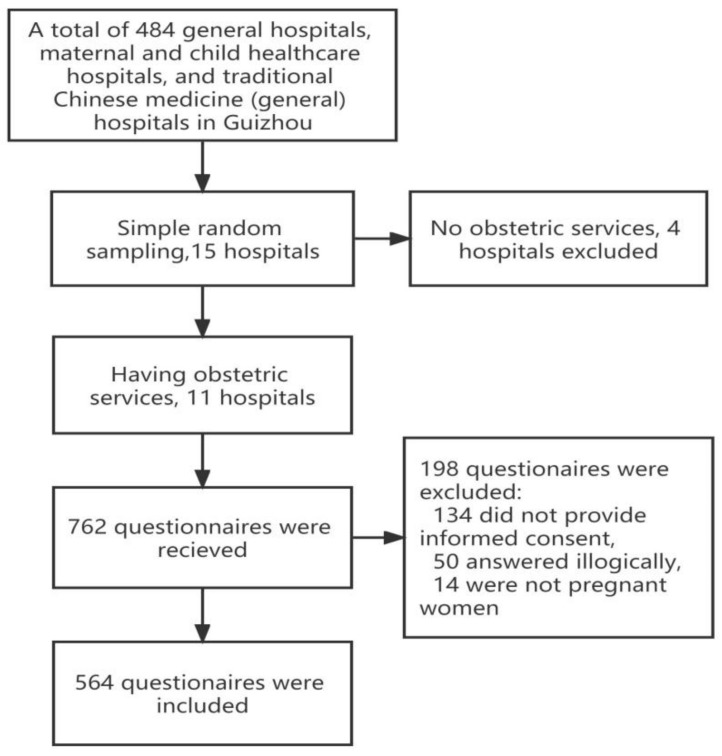
Flow chart detailing processes of the study.

In total, 762 questionnaires were received, of which 198 were excluded (134 did not provide informed consent, 14 did not meet the inclusion criteria of women undergoing pregnancy, and 50 provided illogical answers (26 did not give the correct answers for the specific screening question; 11 made mutually exclusive choices for a multiple choice question; and 13 filled in their birthday incorrectly, which resulted in the age of pregnant participants being less than 8 years of age or more than 100 years of age)). As a result, 564 valid questionnaires were included in the analysis (Figure 1).

### 3.1. Demographic Characteristics 

The pregnant women included through eligible questionnaires had a median age of 27 years (IQR 24–31) and had one child (IQR 0–1). The number of participants with educational levels of middle school or below, high school/technical school, and bachelor’s degree or above was 205 (36.3%), 205 (36.3%), and 154 (27.3%), respectively. The types of residence included city, town, and village, and the number of participants for each was 251 (44.5%), 136 (24.1%), and 177 (31.4%), respectively. In total, 471 (83.51%) were employed in nonmedical institutions and 93 (16.5%) in medical institutions; 36 (6.4%) had pre-existing diseases before pregnancy; and 57 (10.1%) were diagnosed with pregnancy-induced diseases. The time at which they participated in the survey was at 29 pregnancy weeks (IQR 18–36). Within one year before pregnancy, 67 (11.9%) of the pregnant women received the influenza vaccine and 497 (88.1%) either did not receive the vaccine or had an unknown vaccination history (Table 1). 

### 3.2. Epidemiological Knowledge Levels among Pregnant Women

There were six items of common knowledge each for pertussis and influenza, and each of these items could be scored as 0 or 1. The median total score for pertussis epidemiological knowledge was 3 (IQR 1–5), and for influenza, it was 6 (IQR 4–6). The awareness rates for pertussis, comprising knowledge on its infectious nature, the main source of infection, the main mode of transmission, the main clinical manifestation, and relevant vaccination, were 33.7%, 38.3%, 54.1%, 33.7%, and 59.4%, respectively. For influenza, these values were 82.5%, 71.6%, 77.3%, 77.5%, and 77.5%. For each item, the awareness rate of influenza was significantly higher than that of pertussis (71.6–89.2% vs. 33.7–59.4%, *p* < 0.001) (Table 2).

### 3.3. Willingness to Be Vaccinated in Different Life Stages and Contexts

Given a vaccine was available, the types of vaccination-related actions the pregnant women were willing to undertake against pertussis, from most to least, were free vaccination of baby (80.3%), recommend vaccination to family members (71.3%), vaccination for postpartum women (64.5%), vaccination of the baby at their own expense (61.4%), and vaccination during pregnancy (36.0%). For influenza, the results were as follows: recommend vaccination to family members (74.8%), vaccination for postpartum women (67.2%), vaccination of the baby at their own expense (65.8%), and vaccination for pregnant women during pregnancy (36.7%). There were significant differences in the pregnant women’s willingness to be vaccinated in different periods (Friedman test: $\chi^{2}$_pertussis_ = 362.548, *p* < 0.001; $\chi^{2}$_influenza_ = 497.386, *p* < 0.001). The willingness rates to receive vaccinations during pregnancy (pertussis: 36.0%; influenza: 36.7%) were significantly lower than for other periods (*p* < 0.001). Meanwhile, there was no significant difference in the pregnant women’s willingness to be vaccinated against pertussis and influenza in either period (Table 3).

### 3.4. Correlation between Knowledge Levels and Vaccination Willingness

Among the pregnant women who agreed to vaccinations or their recommendation, the knowledge scores were relatively high. The knowledge scores were correlated with the willingness to be vaccinated against pertussis during pregnancy (Spearman’s r = 0.141, *p* = 0.001) and vaccination against influenza during pregnancy (Spearman’s r = 0.083, *p* = 0.048). The correlation coefficient varied from 0.246 for willingness to be postnatally vaccinated against pertussis to 0.407 for willingness to recommend vaccination against influenza to family members (Table 4).

### 3.5. Influential Factors of Knowledge Levels

#### 3.5.1. Univariate Analysis

Substantial differences in the domain of pertussis knowledge status have been identified, namely, in the groups of age ($\chi^{2}$ = 16.783, *p* < 0.001), pregnancy stage ($\chi^{2}$ = 16.299, *p* < 0.001), ethnicity ($\chi^{2}$ = 5.555, *p* < 0.018), educational level ($\chi^{2}\;$= 48.078, *p* < 0.001), occupation ($\chi^{2}$ = 90.181, *p* < 0.001), type of residence ($\chi^{2}$ = 27.462, *p* < 0.001), registered residence ($\chi^{2}$ = 23.354, *p* < 0.001), monthly household income per capita ($\chi^{2}$ = 7.797, *p* = 0.020), and prepregnancy influenza vaccination ($\chi^{2}\;$= 17.401, *p* < 0.001). Furthermore, there were significant differences in influenza knowledge in the groups of age ($\chi^{2}\;$= 9.982, *p* = 0.007), pregnancy stage ($\chi^{2}\;$= 5.944, *p* = 0.050), pregnancy-induced disease ($\chi^{2}$ = 4.268, *p* = 0.039), educational level ($\chi^{2}$ = 41.383, *p* < 0.001), occupation ($\chi^{2}$ = 34.033, *p* < 0.001), type of residence ($\chi^{2}$ = 22.174, *p* < 0.001), registered residence ($\chi^{2}\;$= 16.733, *p* < 0.001), and monthly household income per capita ($\chi^{2}$ = 12.710, *p* = 0.002), whereas there was no significant difference in the groups of the prepregnancy influenza vaccinations ($\chi^{2}$ = 1.830, *p* = 0.176) (Table 5).

#### 3.5.2. Multivariate Analysis of Knowledge Levels

Among the pregnant women, the factors independently influencing the low levels of pertussis knowledge were occupation as nonmedical-institution staff (AOR = 21.64 (95% CI: 7.65–61.23), *p* < 0.001), educational level (middle school and below vs. bachelor’s degree or above, AOR = 2.44 (95% CI: 1.46–4.07), *p* = 0.001; high school/technical school vs. bachelor’s degree or above, AOR = 1.87 (95% CI: 1.13–3.09), *p* = 0.014), pregnancy stage (first trimester vs. third trimester, AOR = 0.42 (95% CI: 0.24–0.76), *p* = 0.004), and not bearing children (AOR = 1.50 (95% CI: 1.02–2.21), *p* = 0.041); and the factors independently influencing the low levels of influenza knowledge were occupation as nonmedical-institution staff (AOR = 2.93 (95% CI: 1.65–5.20), *p* < 0.001), educational level (middle school and below vs. bachelor’s degree or above, AOR = 2.78 (95% CI: 1.70–4.55), *p* < 0.001; high school/technical school vs. bachelor’s degree or above, AOR = 1.70 (95% CI: 1.07–2.71), *p* = 0.025), denial of pregnancy-induced disease (AOR = 1.85 (95% CI: 1.02–3.37), *p* = 0.045), and monthly household income per capita (CNY < 1000 vs. CNY ≥ 5000, AOR = 1.86 (95% CI: 1.00–3.47), *p* = 0.05; CNY 1000–4999 vs. CNY ≥ 5000, AOR = 1.79 (95% CI: 1.07–2.98), *p* = 0.026).

Among the pregnant women working in nonmedical institutions, the factors independently influencing low levels of pertussis knowledge were educational level (middle school and below vs. bachelor’s degree or above, AOR = 2.30 (95% CI: 1.37–3.86), *p* = 0.002; high school/technical school vs. bachelor’s degree or above, AOR = 1.75 (95% CI: 1.05–2.94), *p* = 0.033), pregnancy stage (first trimester vs. third trimester, AOR = 0.44 (95% CI: 0.25–0.80), *p* = 0.007) and minority ethnicity (AOR = 0.67 (95% CI: 0.44–1.00), *p* = 0.049); and the factors independently influencing low levels of influenza knowledge were educational level (middle school and below vs. bachelor’s degree or above, AOR = 3.20 (95% CI: 1.93–5.32), *p* < 0.001; high school/technical school vs. bachelor’s degree or above, AOR = 1.95 (95% CI: 1.16–3.27), *p* = 0.011) and denial of pregnancy-induced disease (AOR = 2.07 (95% CI: 1.10–3.89), *p* = 0.024) (Table 6).

## 4. Discussion

### 4.1. Results Overview

Maternal vaccination was proven to be the most effective method to protect young infants from the serious diseases of pertussis and influenza [3]. Furthermore, women play crucial roles in making vaccination decisions for their babies and recommending other households to vaccinate. Therefore, the vaccination attitudes and choices of pregnant women require special attention. In this survey, a total of 564 valid questionnaires completed by pregnant women were included in the analysis. In total, 11.9 percent of the pregnant women had received the influenza vaccine in the year preceding their pregnancy. This proportion was between that of the group over 65 years of age (2.15%) and the children group, aged six months to five years (16.27%), in Guizhou [23,24]. Regarding the disease common-knowledge level, the score for pertussis was obviously lower than that for influenza (median scores, 3 vs. 6). The pregnant women’s willingness to partake in the following vaccination-related actions (against pertussis and influenza, respectively) could be ranked, from high to low, as free vaccination of baby (only available for pertussis, 80.3%), recommend vaccination to family members (71.3%; 74.8%), postpartum vaccination (64.5%; 67.2%), vaccination of baby at own expense (61.4%; 65.8%), and vaccination during pregnancy (36.0%; 36.7%). Despite the significant difference between pertussis and influenza in terms of knowledge levels, the levels of willingness to vaccinate against pertussis and influenza were similar considering vaccinations in the different life stages and contexts. Knowledge levels played different roles in the willingness in these different situations (Spearman’s r, 0.083–0.407). Additionally, the role of common knowledge was limited for the attitude toward vaccination during pregnancy. 

### 4.2. Knowledge Levels and Influential Factors

The pregnant women in Guizhou province, China demonstrated a higher knowledge of influenza and its vaccine than of pertussis and its vaccine. A total of 10.8% of the pregnant women had never heard of influenza and 22.5% did not know the influenza vaccine can prevent influenza disease, whereas the corresponding figures for pertussis were as high as 46.3% and 40.6%. Each item, including infectious source, transmission mode, susceptible population, and main clinical manifestation for pertussis, scored significantly lower than for influenza. In Guizhou province, the influenza vaccine was one of the optional and self-funded vaccines for all populations. Although the SIV rate was still low, wider communication about influenza and vaccines might lead to increased knowledge and awareness. Contrarily to the seasonal influenza vaccine (SIV), DTP has been included in the National Immunization Program for decades and it is free for children less than six years of age [8]. However, pertussis-containing vaccines applied to people six years of age and above are not available in China [9]. This application background of pertussis-containing vaccines might contribute to the low knowledge and awareness of pertussis and its vaccine. This shortage of pertussis knowledge is common in Asian countries. An investigation among travelers in Singapore in 2006 showed that travelers from Western countries were seven times more likely than Asians to have knowledge about pertussis [27]. Another survey in Singapore showed 92 percent of pregnant women have heard about influenza, but the rate for pertussis was only 42 percent [5]. The early resurgence of pertussis and the change in the immunization strategy might have contributed to the greater awareness of pertussis in Western countries than among Asian countries [28]. The awareness of pertussis and vaccines needs to be increased in Guizhou province, China.

Regarding influencing factors for knowledge levels, occupation in medical institutions and educational levels were similar for both influenza and pertussis. The results were similar to another survey in China that showed respondents’ knowledge levels regarding influenza and influenza vaccination were on an increasing trend as their education levels increased [25]. Compared with the pregnant women working in medical institutions, those working in nonmedical institutions had a more obvious shortage of knowledge on pertussis (AOR = 21.64, 95% CI: 7.65–61.23) than influenza (AOR = 2.93, 95% CI: 1.65–5.20). Moreover, the obvious differences in the influential factors between pertussis and influenza contributed to these variables: for pertussis, pregnancy stage and not bearing children; for influenza, denial of pregnancy-induced diseases and monthly household income per capita. The pregnant women who visited the gynecology and obstetrics clinics in the first trimester of their pregnancy and those bearing children expressed a better understanding of pertussis. Furthermore, those in denial of pregnancy-induced diseases and with high household incomes had a greater understanding about influenza. A survey of the antenatal-care status of pregnant women in mainland China in 2018 showed that the proportion of first antenatal-care examinations in the first trimester was 61.87% [29]. The timing of the first antenatal-care examination visit was shorter in groups with higher levels of education, residents of urban areas, and rich populations [30]. Our results imply that the pregnant women who registered and took antenatal examinations in the first trimester of pregnancy had higher health literacy on pertussis because of their higher educational levels and better socioeconomic situations. The experience of raising children could mean mothers receive more information about pertussis and DTP vaccines for children. Thus, pregnant women bearing children could have higher knowledge levels of pertussis than those not bearing children. Pregnancy-induced diseases, such as gestational diabetes, pre-eclampsia, and pregnancy-associated hypertension, occur more often in women ≥ 40 years of age [31], whereas the survey shows that pregnancy-induced disease was an independent influential factor of the influenza knowledge level. The pregnant women who denied having a pregnancy-induced disease were about twice as likely to have lower influenza knowledge levels. The WHO recommends individuals with comorbidities and underlying conditions as a target group of influenza vaccination [32]. Recommendations on influenza prevention and vaccination from healthcare staff might contribute to increasing knowledge levels and awareness of influenza among pregnant women with pregnancy-induced diseases [33,34]. In addition, household income was a positive influencing factor for influenza knowledge levels, as many previous studies have shown [35].

In the subgroup of pregnant women working in nonmedical institutions, the influencing factors of knowledge levels were similar to those of the main group. The main difference was contributed to by the factors of bearing children and ethnicity for pertussis, and monthly household income per capita for influenza. The confidence limits of factors of bearing children and ethnicity for pertussis were near one. Thus, the influence of bearing children and ethnicity on pertussis knowledge levels and the potential rationales should be explored further. Monthly household income per capita was excluded from the list of influencing factors for influenza knowledge levels in the subgroup. This might contribute to the association between income and occupation. Among pregnant women working in nonmedical institutions, a lower educational level and denial of pregnancy-induced diseases were exclusively the influencing factors for low levels of influenza knowledge.

### 4.3. Vaccine Willingness and Hesitancy

Among the pregnant women, although the influenza-knowledge levels were obviously higher than those of pertussis, the vaccination-willingness levels for influenza were similar to those for pertussis. Meanwhile, prepregnancy influenza vaccination was associated with the knowledge levels for pertussis ($\chi^{2}$ = 17.401, *p* < 0.001), but not for influenza ($\chi^{2}$ = 1.830, *p* = 0.176). Furthermore, the willingness to vaccinate against influenza during pregnancy had the lowest correlation with the knowledge levels (Spearman’s r = 0.083, *p* = 0.048). Hence, compared with pertussis vaccinations, hesitancy toward influenza vaccines might be more serious [36,37]. The willingness to be vaccinated during pregnancy was lower (pertussis vaccine: 36.0% vs. influenza vaccine: 36.7%) than the vaccination willingness in other life stages. The pregnant women’s willingness to receive the pertussis vaccination was similar to that found in a previous survey in Italy [38]. However, the pregnant women’s willingness to receive influenza vaccines was obviously lower than that (50.8%) in Guangzhou City, China [39]. The populations’ educational levels, socioeconomic situations, and so on might contribute to the difference. The rate of the pregnant women’s willingness to vaccinate their babies at their own expense (pertussis vaccine: 61.4%) was the second lowest out of the five conditions for vaccination examined. If pertussis vaccinations were not free, the vaccination-willingness rate for babies would decrease by 18.9 percent, from 80.3 percent to 61.4 percent. Free vaccination is efficacious in decreasing vaccination hesitancy and increasing vaccination rates [40]. In this study, 74.8% of pregnant women had a willingness to recommend influenza vaccination to family households and the rate was similar in a domestic telephone survey (72%) [41]. Some surveys showed that the subjects’ willingness to recommend influenza vaccinations was usually much lower than their own influenza vaccination rates [42]. Compared with the willingness for family-household vaccination, the willingness for postnatal vaccination (pertussis vaccine: 64.5% vs. influenza vaccine: 67.2%) was lower by 6.8 percent and 7.6 percent, respectively, among the pregnant women. Despite these hindrances, vaccination during pregnancy and the postpartum vaccination of new mothers to reduce pertussis and influenza infection in infants are recommended in some countries [43]. The immunization strategy was constrained by the vaccines and its application scopes. Based on the vaccination willingness in different life stages and contexts, we call for the improvement of vaccines and await for the vaccines to be given at birth [44].

### 4.4. Limitations

This study had several limitations. First, self-reported influenza vaccination and disease histories may result in recall bias. Second, standard questionnaires are still not found in this field and the questionnaire was self-developed. Thus, it was difficult to compare the knowledge levels with other research. Third, the methods of the self-filled questionnaire and the screening question on historical data could exclude women with less literacy and less historical knowledge. Forth, the effect estimated in the pilot study was based on a low sample size, and thus, further studies should be carried out to confirm the results repetitively. Fifth, the factors influencing vaccination willingness and vaccination rates prior to pregnancy were not included in this article, but we will explore this further in subsequent research.

## 5. Conclusions

Maternal immunization has not been initiated in Guizhou. In total, 11.9 percent of the pregnant women studied received the influenza vaccine in the year before their pregnancy in Guizhou province. The pregnant women’s knowledge levels of pertussis were poorer than their knowledge of influenza. Health education and communication on pertussis and influenza should be spread among pregnant women as well as other populations. If a vaccine is available, pregnant women’s vaccination willingness, from most to least, could be ranked according to the following: free vaccination for babies, recommend vaccination to family members, postpartum vaccination, vaccination of babies at the mothers’ own expense, and vaccination during pregnancy. Knowledge levels played different roles in vaccination willingness at different life stages among the pregnant women. The common knowledge of pertussis and influenza played a limited role in the willingness to receive maternal vaccinations. We call for the improvement of vaccines and vaccines given at birth. Further analyses should be designed and implemented to confirm the results and understand the influencing factors and predictors of maternal vaccination willingness beyond the basis of the knowledge–attitudes–practices theory before the initiation of a maternal vaccination program [45].

## Figures and Tables

**Table 1:** Participant characteristics.

Variables	Median (IQR)/*n* (%)
Age (years)	27 (24–31)
Gestational weeks	29 (18–36)
Bearing children (person)	1 (0–1)
Educational level	
Middle school and below	205 (36.3)
High school/technical school	205 (36.3)
Bachelor’s degree or above	154 (27.3)
Occupation	
Nonmedical-institution staff	471 (83.5)
Medical-institution staff	93 (16.5)
Location in the province	
North	379 (67.2)
Middle or south	185 (32.8)
Type of residence	
City	251 (44.5)
Town	136 (24.1)
Village	177 (31.4)
Registered residence	
Urban	160 (28.4)
Agricultural	404 (71.6)
Ethnicity	
Minority	187 (33.2)
Han	377 (66.8)
Pre-existing diseases	36 (6.4)
Pregnancy-induced diseases	57 (10.1)
Monthly household income per capita	
CNY < 1000	107 (19.0)
CNY 1000–4999	367 (65.1)
CNY ≥ 5000	90 (16.0)
Prepregnancy flu vaccination	67 (11.9)

**Table 2:** Knowledge of pertussis and influenza prevention and control among pregnant women *n* (%).

Items (Good Answers)	Scores		Influenza			McNemar *χ*^2^	*p*
			0	1	Total		
Have you heard of it? (yes)	pertussis	0	49 (8.7)	212 (37.6)	261 (46.3)	176.790	<0.001
		1	12 (2.1)	291 (51.6)	303 (53.7)		
		total	61 (10.8)	503 (89.2)	564 (100)		
Is it an infectious disease? (yes)	pertussis	0	80 (14.2)	293 (52.0)	373 (66.1)	238.875	<0.001
		1	19 (3.4)	172 (30.5)	191 (33.9)		
		total	99 (17.6)	465 (82.5)	564 (100)		
What is the main source of infection? (pertussis patients; influenza patients)	pertussis	0	152 (27.0)	196 (34.8)	348 (61.7)	171.417	<0.001
		1	8 (1.4)	208 (36.9)	216 (38.3)		
		total	160 (28.4)	404 (71.6)	564 (100)		
What is the mode of transmission? (respiratory droplet transmission)	pertussis	0	111 (19.7)	148 (26.2)	259 (45.9)	102.424	<0.001
		1	17 (3.0)	288 (51.1)	305 (54.1)		
		total	128 (22.7)	436 (77.3)	564 (100)		
What is the main clinical manifestation? (paroxysmal spasmodic coughing; fever and cough)	pertussis	0	124 (22.0)	250 (44.3)	374 (66.3)	239.194	<0.001
		1	3 (0.5)	187 (33.2)	190 (33.7)		
		total	127 (22.5)	437 (77.5)	564 (100)		
What kind of vaccine can be given to prevent it? (DPT vaccine; influenza vaccine)	pertussis	0	112 (19.9)	117 (20.7)	229 (40.6)	77.280	<0.001
		1	15 (2.7)	320 (56.7)	335 (59.4)		
		total	127 (22.5)	437 (77.5)	564 (100)		

**Table 3:** Attitudes of pregnant women toward pertussis and influenza vaccination in different life stages and contexts.

Items	Pertussis Vaccination (%)			Influenza Vaccination			Wilcoxon Signed-Rank Test	
	Disagree	Unsure	Agree	Disagree	Unsure	Agree	Z	*p*
If a vaccine was available, would agree to receive the vaccine during pregnancy?	143 (25.4)	218 (38.7)	203 (36.0)	149 (26.4)	208 (36.9)	207 (36.7)	−0.085	0.932
After the baby is born, will you vaccinate the baby at your own expense?	53 (9.4)	165 (29.3)	346 (61.4)	57 (10.1)	136 (24.1)	371 (65.8)	−1.153	0.249
If a vaccine was available, would you agree to vaccinate postnatally?	31 (5.5)	169 (30.0)	364 (64.5)	32 (5.7)	153 (27.1)	379 (67.2)	−1.188	0.235
If a vaccine was available, would you recommend a family member to receive it?	12 (2.1)	150 (26.6)	402 (71.3)	13 (2.3)	129 (22.9)	422 (74.8)	−1.729	0.084
After the baby is born, will you allow the baby to be vaccinated for free?	5 (0.9)	106 (18.8)	453 (80.3)	-	-	-		
*χ*^2^ *	362.548			497.386				
*p*	<0.001			<0.001				

* Friedman test.

**Table 4:** Correlation between knowledge scores and vaccination willingness.

Vaccination Willingness		Pertussis-Knowledge Score				Influenza-Knowledge Score			
		N	Mean (SD)	Spearman’s r	*p*	N	Mean (SD)	Spearman’s r	*p*
Agree to be vaccinated during pregnancy	No	143	2.8 (2.1)	0.141	0.001	149	5.0 (1.6)	0.083	0.048
	Unsure	218	2.1 (2.1)			208	4.2 (2.0)		
	Yes	203	3.4 (2.1)			207	5.2 (1.4)		
Agree to vaccinate baby at own expense	No	53	2.7 (2.0)	0.283	<0.001	57	4.4 (2.0)	0.316	<0.001
	Unsure	165	1.6 (1.9)			136	3.7 (2.0)		
	Yes	346	3.3 (2.1)			371	5.2 (1.4)		
Agree to be vaccinated postnatally	No	31	2.9 (1.9)	0.246	<0.001	32	4.1 (2.1)	0.311	<0.001
	Unsure	169	1.8 (1.9)			153	3.9 (2.1)		
	Yes	364	3.2 (2.2)			379	5.2 (1.4)		
Recommend your family member to be vaccinated	No	12	3.0 (1.8)	0.277	<0.001	13	2.9 (2.2)	0.407	<0.001
	Unsure	150	1.7 (1.9)			129	3.6 (2.1)		
	Yes	402	3.1 (2.1)			422	5.2 (1.4)		
Agree to give baby free vaccination	No	5	1.4 (1.1)	0.315	<0.001				
	Unsure	106	1.4 (1.8)						
	Yes	453	3.1 (2.1)						

**Table 5:** Univariate analysis on demographic characteristics and knowledge scores of pertussis and influenza among pregnant women.

Variables	Pertussis-Knowledge Score *n* (%)				Influenza-Knowledge Score *n* (%)			
	0–2	3–6	*χ* ^2^	*p*	0–5	6	*χ* ^2^	*p*
Age (years)								
≤24	102 (62.2)	62 (37.8)	16.783	<0.001	95 (57.9)	69 (42.1)	9.982	0.007
25–29	107 (46.5)	123 (53.5)			106 (46.1)	124 (53.9)		
≥30	69 (40.6)	101 (59.4)			70 (41.2)	100 (58.8)		
Pregnancy Stages								
First trimester	24 (31.6)	52 (68.4)	16.299	<0.001	27 (35.5)	49 (64.5)	5.944	0.050
Second trimester	85 (45.5)	102 (54.5)			90 (48.1)	97 (51.9)		
Third trimester	169 (56.1)	132 (43.9)			154 (51.2)	147 (48.8)		
Bearing children								
No	126 (53.8)	108 (46.2)	3.320	0.068	117 (50.0)	117 (50.0)	0.609	0.435
Yes	152 (46.1)	178 (53.9)			154 (46.7)	176 (53.3)		
Pre-existing diseases								
No or unknown	263 (49.8)	265 (50.2)	0.894	0.344	253 (47.9)	275 (52.1)	0.059	0.809
Yes	15 (41.7)	21 (58.3)			18 (50.0)	18 (50.0)		
Pregnancy-induced diseases								
No or unknown	249 (49.1)	258 (50.9)	0.064	0.801	251 (49.5)	256 (50.5)	4.268	0.039
Yes	29 (50.9)	28 (49.1)			20 (35.1)	37 (64.9)		
Ethnicity								
Minority	79 (42.2)	108 (57.8)	5.555	0.018	86 (46.0%)	101 (54.0)	0.476	0.490
Han	199 (52.8)	178 (47.2)			185 (49.1)	192 (50.9)		
Educational level								
Middle school and below	133 (64.9)	72 (35.1)	48.078	<0.001	130 (63.4)	75 (36.6)	41.383	<0.001
High school/technical school	102 (49.8)	103 (50.2)			96 (46.8)	109 (53.2)		
Bachelor’s degree or above	43 (27.9)	111 (72.1)			45 (29.2)	109 (70.8)		
Occupation								
Nonmedical-institution staff	274 (58.2)	197 (41.8)	90.181	<0.001	252 (53.5)	219 (46.5)	34.033	<0.001
Medical-institution staff	4 (4.3)	89 (95.7)			19 (20.4)	74 (79.6)		
Location in the province								
North	185 (48.8)	194 (51.2)	0.106	0.745	174 (45.9)	205 (54.1)	2.119	0.146
Middle or south	93 (50.3)	92 (49.7)			97 (52.4)	88 (47.6)		
Type of residence								
City	95 (37.8)	156 (62.2)	27.462	<0.001	94 (37.5)	157 (62.5)	22.174	<0.001
Town	71 (52.2)	65 (47.8)			71 (52.2)	65 (47.8)		
Village	112 (63.3)	65 (36.7)			106 (59.9)	71 (40.1)		
Registered residence								
Urban	53 (33.1)	107 (66.9)	23.354	<0.001	55 (34.4)	105 (65.6)	16.733	<0.001
Agriculture	225 (55.7)	179 (44.3)			216 (53.5)	188 (46.5)		
Monthly household Income per capita								
CNY < 1000	65 (60.7)	42 (39.3)	7.797	0.020	61 (57.0)	46 (43.0)	12.710	0.002
CNY 1000–4999	175 (47.7)	192 (52.3)			181 (49.3)	186 (50.7)		
CNY ≥ 5000	38 (42.2)	52 (57.8)			29 (32.2)	61 (67.8)		
Prepregnancy flu vaccination								
No/unknown	261 (52.5)	236 (47.5)	17.401	<0.001	244 (49.1)	253 (50.9)	1.830	0.176
Yes	17 (25.4)	50 (74.6)			27 (40.3)	40 (59.7)		

**Table 6:** Multivariate analysis of epidemiological knowledge levels of pertussis and influenza among pregnant women.

Dependent	Variables	Pregnant Women (*n* = 564)			Pregnant Women Working in Nonmedical Institutions (*n* = 471)		
		AOR	95% CI	*p*	AOR	95% CI	*p*
Knowledge level of pertussis *	Educational level			0.003			0.007
	Middle school and below	2.44	(1.46, 4.07)	0.001	2.30	(1.37, 3.86)	0.002
	High school/technical school	1.87	(1.13, 3.09)	0.014	1.75	(1.05, 2.94)	0.033
	Bachelor’s degree or above	1.0			1.0		
	Pregnancy Stages			0.012			0.021
	First trimester (≤13 weeks)	0.42	(0.24, 0.76)	0.004	0.44	(0.25, 0.80)	0.007
	Second trimester (14–27 weeks)	0.73	(0.48, 1.10)	0.135	0.75	(0.49, 1.14)	0.178
	Third trimester (28–40 weeks)	1.0			1.0		
	Bearing children						
	No	1.50	(1.02, 2.21)	0.041	1.44	(0.98, 2.14)	0.067
	Yes	1.0			1.0		
	Ethnicity						
	Minority	0.72	(0.48, 1.07)	0.104	0.67	(0.44, 1.00)	0.049
	Han	1.0					
	Occupation						
	Nonmedical-institution staff	21.64	(7.65, 61.23)	<0.001			
	Medical-institution staff	1.0					
Knowledge level of influenza ^#^	Educational level			<0.001			<0.001
	Middle school and below	2.78	(1.70, 4.55)	<0.001	3.20	(1.93, 5.32)	<0.001
	High school/technical school	1.70	(1.07, 2.71)	0.025	1.95	(1.16, 3.27)	0.011
	Bachelor’s degree or above	1.0			1.0		
	Pregnancy-induced diseases						
	No or unknown	1.85	(1.02, 3.37)	0.045	2.07	(1.10, 3.89)	0.024
	Yes	1.0			1.0		
	Monthly household income per capita			0.070			
	CNY < 1000	1.86	(1.00, 3.47)	0.050			
	CNY 1000–4999	1.79	(1.07, 2.98)	0.026			
	CNY ≥ 5000	1.0					
	Occupation						
	Nonmedical-institution staff	2.93	(1.65, 5.20)	<0.001			
	Medical-institution staff	1.0					

AOR: adjusted odds ratio; 95% CI: 95% confidence interval. * Knowledge level of pertussis, 1: “0–2 scores”; 0: “3–6 scores”. A total of ten variables (age, pregnancy stage, bearing children, pregnancy-induced diseases, ethnicity, educational level, occupation, location in the province, type of residence, monthly household income per capita) were included in the binary logistic regression in the pregnant women group (*n* = 564). For the pregnant women working in nonmedical institutions (*n* = 471), the variables above were screened (except the variable of occupation). ^#^ Knowledge level of influenza, 1: ”0–5 scores”; 0: “6 scores”. A total of seven variables (age, pregnancy stage, pregnancy-induced diseases, educational level, occupation, type of residence, monthly household income per capita) were included in the binary logistic regression in the pregnant women group (*n* = 564). For the pregnant women working in nonmedical institutions (*n* = 471), the variables above were screened (except the variable of occupation).

## Data Availability

Not applicable due to privacy reasons.
